# Comorbid mental disorders in substance users from a single catchment area - a clinical study

**DOI:** 10.1186/1471-244X-11-25

**Published:** 2011-02-12

**Authors:** Anne-Marit Langås, Ulrik F Malt, Stein Opjordsmoen

**Affiliations:** 1 Vestre Viken Hospital Trust, Kongsberg, Norway; 2 University of Oslo, Institute of Clinical Medicine, Oslo, Norway; 3 Oslo University Hospital, Oslo, Norway; 4 Norwegian Research Network on Mood Disorders (NORMOOD), Oslo, Norway

## Abstract

**Background:**

The optimal treatment of patients with substance use disorders (SUDs) requires an awareness of their comorbid mental disorders and vice versa. The prevalence of comorbidity in first-time-admitted SUD patients has been insufficiently studied. Diagnosing comorbidity in substance users is complicated by symptom overlap, symptom fluctuations, and the limitations of the assessment methods. The aim of this study was to diagnose all mental disorders in substance users living in a single catchment area, without any history of treatment for addiction or psychiatric disorders, admitted consecutively to the specialist health services. The prevalence of substance-induced versus substance-independent disorders according to the Diagnostic and Statistical Manual of Mental Disorders, Fourth Edition (DSM-IV), in SUD patients will be described.

**Methods:**

First-time consecutively admitted patients from a single catchment area, aged 16 years or older, admitted to addiction clinics or departments of psychiatry as outpatients or inpatients will be screened for substance-related problems using the Alcohol Use Disorder Identification Test and the Drug Use Disorder Identification Test. All patients with scores above the cutoff value will be asked to participate in the study. The patients included will be diagnosed for SUD and other axis I disorders by a psychiatrist using the Psychiatric Research Interview for Substance and Mental Disorders. This interview was designed for the diagnosis of primary and substance-induced disorders in substance users. Personality disorders will be assessed according to the Structured Clinical Interview for DSM-IV axis II disorders. The Symptom Checklist-90-Revised, the Inventory of Depressive Symptoms, the Montgomery Asberg Depression Rating Scale, the Young Mania Rating Scale, and the Angst Hypomania Check List will be used for additional diagnostic assessments. The sociodemographic data will be recorded with the Stanley Foundation's Network Entry Questionnaire. Biochemical assessments will reveal somatic diseases that may contribute to the patient's symptoms.

**Discussion:**

This study is unique because the material represents a complete sample of first-time-admitted treatment seekers with SUD from a single catchment area. Earlier studies have not focused on first-time-admitted patients, so chronically ill patients, may have been overrepresented in those samples. This study will contribute new knowledge about mental disorders in first-time-admitted SUD patients.

## Background

### Burden of comorbid disorders

The high frequency of comorbid mental disorders in individuals with a high intake of psychoactive substances has been well documented in clinical and epidemiological studies. Such dual disorders are a matter of great concern because of their serious consequences for the patients, their families, health services, and society. Compared with patients diagnosed with a single mental disorder or substance use disorder (SUD), patients with comorbid disorders run a higher risk of delayed diagnosis [1], more severe psychopathological symptoms [2], less compliance with treatment [3], poorer effects of treatment [4], more impairment of social functioning [5], increased admissions to emergency departments [6], higher prevalence of physical comorbidity [7], and suicidal ideation [8]. They are also more often unemployed [9], homeless [10], and involved in violent episodes [11] or criminal behavior [12]. The poor outcomes of these patients call for more research within this field.

### Necessity of diagnosing comorbid disorders

Traditionally, SUDs and psychiatric disorders have been treated as separate conditions. However, in the last few decades, the close connection between the two has been increasingly acknowledged [13]. Attention has focused on the fact that many psychiatric patients have undiagnosed comorbid SUDs, which go untreated, and therefore jeopardize the treatment of their mental disorder. The majority of SUD patients also have mental disorders, and often do not receive the appropriate treatment.

Many patients receive treatment from both mental and addiction services, but these are uncoordinated and are given at different times. Patients are sometimes rejected in one kind of clinic and sent to another, based on the disorder that is considered to be their major problem. Comorbid disorders may be treated sequentially, simultaneously, or in an integrated way, depending on the type and severity of the two disorders. Integrated treatments are now commonly recommended for more severe disorders [14-16]. Some treatment modalities may be the treatment of choice for both the mental and substance-related disorders; e.g., cognitive behavioral therapy or medication.

The reliable assessment of comorbid disorders is usually achieved in non-SUD psychiatric patients with one or more comorbid psychiatric disorders, in patients with a mental disorder and a previous but not ongoing SUD, and in patients with only an SUD diagnosis. It is much more complicated to assess the mental disorders of patients with ongoing SUD [17]. A successful diagnosis is essential for well-adapted and high-quality treatment. Therefore, it is extremely important that all the disorders of a patient are diagnosed.

Earlier studies have shown variations in the prevalence of comorbid substance use and mental disorders. This is attributable to a lack of consensus regarding the definition of the term "comorbidity", problems in distinguishing induced and independent disorders, problems in separating psychiatric disorders from the symptoms of intoxication or withdrawal, the choice of diagnostic instruments, the skills of the interviewers, and differences in the study samples. In most studies, patients are recruited from general populations or selected from clinical treatment units. As far as we know, no previous study has included all possible subjects from a single catchment area within a specific time period.

### Classification of disorders related to the use of psychoactive substances

When patients are heavy users of psychoactive substances, it is challenging to assess their psychiatric symptoms, which may be independent of their substance use, caused by intoxication or withdrawal, or an expected effect of the substance used. The Diagnostic and Statistical Manual of Mental Disorders, Fourth Edition (DSM-IV) of the American Psychiatric Association distinguishes "Substance use disorders" (SUDs), i.e., dependence on or abuse of a psychoactive substance, and "Substance-induced disorders" (SIDs), which are mental disorders caused by substance use, i.e., occurring during a period of heavy use or during the first four weeks of withdrawal. To diagnose an SID, the substance must be known to cause the type of symptoms observed, and the symptoms must be in excess of the expected effect of the substance. Such symptoms should not be diagnosed as symptoms of a primary psychiatric disorder, even if the symptoms of the two conditions are identical. The symptoms of an SID must be sufficiently severe to warrant independent clinical attention. An SID does not always need to fulfill all the diagnostic criteria of the related primary psychiatric disorder. The diagnoses of dependence, abuse, intoxication, and withdrawal for each substance are described in the chapter entitled "Substance-Related Disorders" in DSM-IV. Other substance-induced disorders, such as delirium, psychotic disorder, mood disorder, and anxiety disorder, are described in the chapters concerning the respective mental disorders. DSM-IV does not include substance-induced personality disorders.

### Relationship between mental disorders and substance use

There are several types of relationships between mental disorders and SUDs. The causes of comorbidity may include coincidence, common genetic vulnerability, common neural substrate, underlying shared origins, self-medication, environment, and lifestyle.

The terms "primary" and "secondary" are frequently applied to disorders in the literature. "Primary" refers to the first condition to develop. This is a chronologically based term only, and does not necessarily represent causality. More meaningfully, it should be recognized that some disorders are independent and some are induced by other disorders [18,19]. Most patients with SUD report that their symptoms of a mental disorder preceded their SUD. In some cases, this may mean that their mental symptoms caused their SUD (e.g., the self-medication hypothesis) [20]. In other cases, it may indicate that the age of onset of some mental disorders is lower than the age of onset of an SUD [21]. Some symptoms of mental disorders are temporary, caused by substance intoxication or withdrawal [22,23]. For instance, the high incidence of depression in SUD patients may represent such a phenomenon, and this is sometimes called the "substance-related artifact hypothesis" [24]. However, depressive symptoms in addicted patients may reflect neuroadaptations in the dopamine system caused by chronic drug administration [25,26]. Drug-induced changes in neurotransmitter systems alter the function of the reward circuitry [27] and motivational and behavioral systems in the brain [28,29]. This causes symptoms such as dysthymia, anhedonia, irritability, and motivational and emotional changes during drug withdrawal.

As mentioned above, some mental disorders are coincidentally concurrent with substance abuse. Both disorders may then run their different courses, or they might exacerbate the prognosis of the other. The high frequency of comorbidity reflects the overlapping environmental, genetic, and neurobiological factors that negatively influence both types of disorders. Early life stress or chronic stress results in long-term changes in stress responses, which may alter the sensitivity of the dopamine system. Low dopamine activity makes the individual susceptible to the self-administration of drugs. The chronic stress model suggests why the substance abuse of some susceptible patients increases their risk of mental disorders and vice versa [30].

Substance abuse or dependence develops in the course of repeated substance use. The amount of substance necessary varies with age, genetics, and other risk factors. In adolescents, the brain regions involved in the process of executive control and motivation are still incompletely developed. Therefore, repeated drug use in adolescents leads to long-lasting brain changes, which undermine voluntary control, hinder brain maturation, and make the brain susceptible to the development of further SUDs. Early drug use is associated with, and predicts, later mental disorders [31,32].

To distinguish between independent and substance-induced disorders, the following questions should be answered. 1) Are the symptoms in excess of those expected given the type and amount of substance used and the duration of use? 2) Have the symptoms occurred in periods of abstinence? 3) Did the onset of the symptoms precede the onset of the substance use by a sufficient time period? 4) Have the symptoms persisted for at least a month after the cessation of substance use? 5) Does any close relative of the patient have the same or a related disorder? 6) Can the symptoms be explained by a medical condition or the treatment of such a condition? 7) Can the symptoms be explained by exposure to other noxious agents?

### Diagnostic challenges in epidemiological studies

In epidemiological studies of comorbid mental disorders and SUDs, there is a risk of exaggerating both the number of SUD diagnoses and the number of primary mental diagnoses. In such studies, patients with symptoms that do not meet all the criteria for the diagnostic categories may be included. Moreover, symptoms related to drug abuse (e.g., nervousness, tension, agitation, depressed mood, loss of motivation) may at the same time be symptoms included in the diagnostic criteria for mental disorders (e.g., generalized anxiety disorder, depressive disorder). In epidemiological studies, persons with SUDs are in different stages of their disorder: intoxicated, abstinent, or experiencing withdrawal symptoms. Moreover, the most severely ill patients are probably unable to participate in the surveys.

Research instruments are also often insufficiently sensitive to discriminate between independent and substance-induced symptoms in patients with ongoing substance abuse, intoxication, or withdrawal symptoms. The traditional use of trained but nonprofessional interviewers may be a problem when clinical judgments are required. Caution is needed in interpreting the results of many studies, because the diagnoses were made by nonclinicians and the symptoms were reported retrospectively.

Studies within this field have methodological problems related to the differentiation of alcohol/drug abuse and other mental disorders. In some studies, diagnoses are based only on screening instruments; in others, the diagnostic interviews used have not been validated for both SUDs and mental disorders. Diagnoses drawn from different diagnostic interviews give different results, to varying extents [33-35], and even the same diagnostic interview, used in different groups, can poorly differentiate psychiatric symptoms from symptoms of intoxication or withdrawal [36]. Structured instruments have been shown to increase the diagnostic validity of SUD diagnoses compared with clinical judgments, but psychiatric comorbid diagnoses show poor validity, regardless of the method used [37,38].

### Prevalence of comorbidity in epidemiological studies

Epidemiological studies from different countries have shown a high prevalence of comorbid alcohol or other drug disorders and mental disorders. In the Epidemiologic Catchment Area Program in the USA undertaken in the early 1980s, the estimated prevalence of mental disorders was 22.5%, and the lifetime incidence was 32%. Among subjects with an alcohol disorder, 37% had a comorbid mental disorder, and among drug-abusing subjects, 53% had a mental disorder. In the general population, 16% of individuals had an SUD, whereas 29% of people with a mental disorder had a comorbid SUD [39].

The US National Comorbidity Study (NCS) undertaken in the late 1980s found that almost 50% of the participants met the criteria for at least one lifetime mental disorder, and almost 30% had suffered at least one mental illness in the preceding year. The most common disorders were severe depression, compulsive drinking, and social and simple phobias. Among male alcoholics, 78% had a comorbid mental disorder or SUD, as did 86% of female alcoholics [40,41]. The overall results from the NCS are very similar to those obtained with a Norwegian epidemiological study in Oslo [42], except for a lower prevalence of illegal drug abuse in Norway. A similar study performed in a rural area in western Norway showed a lower prevalence of all disorders, but the same basic pattern was observed [43].

The European Study of the Epidemiology of Mental Disorders was a population study performed in six European countries between 2001 and 2003 [44]. Among all the subjects, 14% reported a lifetime history of a mood disorder, 13.6% an anxiety disorder, and 5.2% an alcohol disorder. Mental disorders were more frequent in younger people and in female, unemployed, unmarried, or disabled people.

In a Canadian study, the 12-month prevalence of major depressive disorder (MDD) was almost three times higher in people with substance dependence than in the general population. The risk of MDD and suicidal thoughts increased with more severe substance use [45]. Significant comorbidity between mental disorders and SUDs has been observed in several countries, including the Netherlands [46,47], England [48], Finland [49], Taiwan [50], and Russia [51].

### Diagnostic challenges in clinical samples

In many studies of samples of SUD inpatients, the duration of abstinence before the mental disorder is diagnosed has not been described, or the studies vary in the duration of abstinence examined. Some authors have found that most substance-induced depression and anxiety symptoms decline rapidly with abstinence [52,53]. In most situations, DSM-IV recommends four weeks abstinence before the diagnosis of a mental disorder, to avoid confounding symptoms of intoxication or withdrawal. However, many dependent patients experience a protracted abstinence syndrome, which can last for several months or more. The duration of abstinence required for the symptoms of intoxication or withdrawal to decline varies with the type of substance, the duration of substance use, and the type of symptoms in question [22,54]. In clinical situations with short hospitalizations or nonhospitalized patients, it is often impossible to achieve four weeks of abstinence.

Few studies have been undertaken in outpatient settings with SUD patients, probably because the patients often drop out and are seldom consistently abstinent during the assessment period. It would be very interesting to investigate the possibility of diagnosing nonabstinent individuals in an outpatient clinical setting, because this is the everyday challenge of many clinicians.

Different diagnostic interviews have advantages and limitations when used to assess comorbid SUDs and mental disorders [18,55]. The Psychiatric Research Interview for Substance and Mental Disorders (PRISM) was designed to correct the lack of diagnostic interviews suitable for such assessments [56].

### Prevalence of SUDs in patients with mental disorders

Many studies have demonstrated a high prevalence of SUDs in patients with mental disorders, e.g., in general patient samples [57,58], and in patients with psychoses or schizophrenia [59,60], bipolar disorder [61], depression or anxiety [62], personality disorders [63], and eating disorders [64].

Several studies from acute psychiatric wards in Norway found that about 45% of patients had substance-related problems. Among patients with first-episode nonaffective psychosis in Norway and Denmark, 23% had abused drugs and 15% had abused alcohol during the preceding six months [65]. In another study of psychotic inpatients, 54% had abused substances within the 30 days preceding their admission [66]. It is estimated that between 40% and 50% of patients with psychotic disorders in Western countries also have substance-related disorder. Up to 69% of patients with bipolar disorders have a lifetime history of substance abuse or dependence [61].

In a nationwide Norwegian study, the substance disorder diagnoses of psychiatric inpatients (November 2003) and psychiatric outpatients (September 2004) were registered [67]. Of the patients in psychiatric wards, 10% were diagnosed with dual disorders. The real number is probably higher, because therapists often do not perform exact substance-use assessments.

### Prevalence of mental disorders in SUD patients

A high level of comorbidity between substance abuse and psychiatric disorders in clinical samples has been reported in several countries, including the USA [68-70], Germany [71], Iceland [72], the United Kingdom [73], and New Zealand [74]. The most common psychiatric disorders in SUD patients are anxiety disorders, mood disorders, and personality disorders. Many also have more than one SUD. Much research has been undertaken in this field.

The estimated prevalence of panic disorder and agoraphobia in patients with alcohol use disorders ranges from 5% to 42% [75]. The prevalence of panic disorder varies with the substance used, and only 1.7% of cocaine-dependent patients experienced panic disorder in a large study [69]. Some symptoms of generalized anxiety disorder (GAD) largely overlap those of acute intoxication with stimulants or withdrawal from alcohol, sedative/hypnotics, or opiates. Therefore, it is not surprising that the prevalence of GAD in different studies varies between 8% and 53% in alcohol-dependent individuals. The same variance has been observed in comorbid alcoholism and social phobia [75]. In cocaine-dependent individuals, social phobia has a lifetime prevalence of 14% [76]. Substance users have a high prevalence of posttraumatic stress disorder of 36%-50% [77]. MDD has been found in 16.5% of patients with alcohol use disorder and in 18% of patients with drug use disorder. Depressive disorder in treatment-seeking alcoholic individuals ranges from 15% to 67% [78]. The prevalence of affective disorders ranges from 33% to 53% in cocaine-dependent individuals and from 16% to 75% in opiate-dependent individuals. In studies of treated addicts, 45% to 80% of the patients had personality disorders [79]. In a study of 370 SUD patients in the USA, 57% had one or more personality disorders, most often in cluster B (45.7%) [80].

In a clinical sample of SUD patients in Norway, 90% had at least one lifetime substance-independent mental disorder, most often an axis I disorder [81]. Furthermore, 79% of polysubstance abusers and 66% of alcohol abusers had one or more axis II disorders.

In a nationwide study in Norway [67], only 25% of patients undergoing treatment for SUD were assessed for a psychiatric disorder, and 65% of the patients presenting with psychiatric problems were not diagnosed. Of the diagnosed patients, 47% had a nonsubstance-related psychiatric disorder, most often an anxiety disorder (34%), mood disorder (25%), or personality disorder (22%).

The wide range of results within diagnostic groups probably does not reflect real differences in prevalence but demonstrates the complexity of reliable assessment.

## Aims

The main aim of this study is to diagnose all mental disorders in substance users from a single catchment area, without any history of treatment for addiction or psychiatric disorder, consecutively admitted to the specialist health services. Because this sample represents patients in whom problematic substance use is identified for the first time, i.e., not readmitted or chronically ill patients, we expect to find a lower prevalence of psychiatric disorders than in previous studies. Because the inpatients and outpatients will be recruited from both addiction treatment clinics and psychiatric treatment clinics, a complete sample of first-time-admitted patients from a single catchment area can be identified and included in the study. To the best of our knowledge, this has not been done in previous studies.

The prevalence of axis I disorders in SUD patients will be described, with special emphasis on whether the disorders are independent mental disorders or induced by substance use. The prevalence of different personality disorders in the sample will also be studied. The time from the first diagnosis of abuse or dependence until the first admission for treatment-the duration of untreated substance use disorder (DUSUD)-will be calculated.

The sample can be divided into two groups that can be compared: patients who use alcohol only and patients who also use illegal drugs. There may be differences in the types and numbers of comorbid disorders, sociodemographic data, or DUSUD between the groups.

### Research questions

1. Which mental disorders are found, and what is their prevalence and severity, when comorbidity is studied in all first-time-admitted substance users consecutively admitted to specialist health services from a single catchment area?

2. What is the average time from the onset of an SUD until the patient's first admission for treatment, the DUSUD?

3. What is the prevalence of substance-induced versus substance-independent depression and other axis I disorders in SUD patients?

4. Are there any differences in mental disorder diagnoses of patients using legal substances and patients using illegal substances?

5. Are there any differences in the sociodemographic data of patients using legal substances and those using illegal substances?

## Methods/Design

### Design

The first part of the study will be a naturalistic cross-sectional descriptive diagnostic study of a clinical sample. The second part will be a comparative study of the two main groups of patients with SUD: (i) patients with alcohol use disorder only, and (ii) patients with SUD caused by psychoactive substances other than alcohol.

### Sample

The patients will be substance users, admitted for the first time as inpatients or outpatients for specialist addiction treatment or specialist psychiatric treatment, from a single catchment area in Norway. In order to ensure that the patients have not previously been treated in the specialist health services, the patients will be thoroughly asked about treatment history, and also asked for written consent to give access to previous medical records. If the patient has been treated elsewhere, he/she will be offered help to find out whether the treatment was on a specialized level (i.e. treatment where a specialized psychologist or physician is responsible). Previous treatment before the age of 16 will be accepted.

The home address of the patients must be in the local hospital area of Kongsberg, which has about 50,000 inhabitants. This region consists of a large rural area, one town with 18,600 inhabitants, and some villages. The distance from one end of the area to the other is about 150 km. There is only one outpatient addiction clinic and one psychiatric inpatient/outpatient clinic in the catchment area. Some patients are referred to psychiatric hospitals or addiction inpatient institutions elsewhere. These institutions will be asked to identify patients who meet the inclusion criteria and to ask them for their consent for inclusion in the project. The youngest patients will be found in child and adolescent psychiatry centers. It may be possible to include all patients admitted for the first time for inpatient addiction treatment because we have an intimate knowledge of, and contact with, all the communities and clinics working with the target group. All possible subjects, aged 16 years and older and admitted consecutively during a specific time period, will be identified by their therapists, who will supply information and ask for the patient's written consent for referral to the study. The time period must be sufficiently long to recruit a representative sample, with a minimum of 60 patients. The substances included in this study will be alcohol, legally prescribed drugs with misuse potential, and illegal drugs.

To identify all the patients with substance-related disorders in psychiatric inpatient and outpatient clinics, all the patients will be screened with the Alcohol Use Disorders Identification Test (AUDIT), with a cutoff of 8 points for men and 6 points for women [82-84]. Those who state that they have tried illegal drugs will be screened with the Drug Use Disorders Identification Test (DUDIT), with a cutoff of 6 points for men and 2 points for women [85]. These cutoffs were chosen because they have been commonly used in other studies. Those patients who use prescribed drugs with misuse or dependence potential will complete the DUDIT. To collect the same data from all the patients included, the patients from addiction clinics will also be screened with the AUDIT and DUDIT. The relatively low cutoffs on these tests have been chosen to increase the sensitivity of the tests, to ensure that all SUD patients are identified.

The patients included must be able to consent to, and cooperate in, the study. Patients with acute intoxication, acute withdrawal symptoms, or acute psychosis will be assessed when the acute symptoms have declined sufficiently for the patient to be able to give reliable information. However, it is not necessary for the patients to be abstinent for a certain period of time or to be completely without psychosis or withdrawal symptoms. If the assessment of a patient gives reason to suspect that he/she has an organic brain disorder, this will also be examined. When referring a patient with suspected organic brain disorder, to further neurological or neuropsychological assessment, the patient will be asked for written consent to receive the results from such assessment. If an organic brain disorder makes it difficult for the patient to give reliable information, he/she will be excluded from the study.

As the assessment is comprehensive, and most of the sample probably will be outpatients, the assessment may take several appointments. The following is planned in order to prevent attrition: (i) all patients will be asked how they want to be contacted if they fail to appear to appointments, (ii) they will be contacted for new appointments as long as they express the agreement to new appointments, (iii) the researchers may do the interviews in any suitable places and at times suggested by the patient, (iv) the patients are motivated by the assurance that the results from the assessments will be communicated to them or to their therapist. The results will, with the patients' consent, be written in the medical record where the patient gets his/her treatment. Thereby their therapist can concentrate on the treatment, and not on further assessment. In the analyzes, the patients will be included if they have given enough information. An overview of drop-outs will be provided within limits decided by the Regional Committee for Medical Research Ethics (REK).

### Clinical and psychometric assessments

Sociodemographic and basic data about the patient's general health will be registered with the shortened version of the Stanley Foundation's Network Entry Questionnaire (NEQ). The NEQ has been used in several major studies of mood disorders and also assesses attitudes/stigma issues about mental disorders [86]. The NEQ will also provide information related to the differentiation of bipolar and unipolar depression.

The screening instrument for psychiatric symptoms is the revised version of the 90-question Symptom Check List (SCL-90R) [87]. Studies have shown high sensitivity and moderate specificity for SCL-90 when used as a screening instrument for mental disorders in SUD patients [88,89]. The sum score, the Global Severity Index (GSI), can be used as a measure of overall psychological distress. However, the nine subscales may not be considered to reflect separate independent dimensions [90].

The patients will also be assessed with the Global Assessment of Functioning, split version (GAF-S and GAF-F). The GAF has high reliability when used by trained researchers [91,92]. Ten random patients will be rescored blindly with GAF, using all available data, to ensure its reliability.

The patients' diagnoses will be based on diagnostic interviews supplemented with clinical judgment. The diagnoses will be defined according to the DSM-IV text revision (TR) because the diagnostic interviews used in this study have been developed for this nomenclature. The sample will be interviewed by a trained psychiatrist using the Psychiatric Research Interview for Substance and Mental Disorders for DSM-IV (PRISM). PRISM is a semistructured interview covering current and lifetime diagnoses of abuse and dependence, 20 axis I disorders, and the two most common personality disorders in substance abusers (antisocial and borderline disorders). PRISM was designed to improve the reliability of the diagnosis of comorbid SUD and mental disorders [93,94]. It has shown promising results on reliability tests [56]. The Norwegian version has recently been authorized.

The PRISM interview is expected to be an instrument suitable for the diagnosis of comorbidity in SUD patients. However, clinicians must still combine various instruments to identify disorders not evaluated by PRISM; e.g., most of the personality disorders and attention deficit (hyperactivity) disorder (AD[H]D). ADHD is a common comorbid diagnosis in SUD patients. The ADHD modules from the extended version of the Mini International Neuropsychiatric Interview (M.I.N.I. plus) will be included. M.I.N.I. is a widely used and well-documented diagnostic interview [95].

Because PRISM only assesses two personality disorders (PD), the Structured Clinical Interview for DSM-IV axis II personality disorders (SCID-II) will be administered to diagnose PDs. SCID-II is an instrument with good reliability in the diagnosis of PD [96].

The Inventory of Depressive Symptoms (IDS) will be used to assess the severity of depressive symptoms [97,98], and its reliability and validity are good [99]. The IDS will collect information about symptoms that is also important for differential diagnoses. The Montgomery Asberg Depression Rating Scale (MADRS) will provide further information about the severity of depression [100,101]. The Young Mania Rating Scale (YMRS) [102], which has proven good psychometric properties [103], will be used to rate manic and bipolar symptoms. The interviewers will attend reliability training on IDS, MADRS, and YMRS until they have reached a consensus correlation above 0.7. On the basis of the IDS, MADRS, and YMRS, the severity of the affective disorder will be evaluated using the Clinical Global Impression. The Angst Hypomania Check List (HCL-32) provides information about previous episodes of possible hyperactivity and hypomania, and is useful as a screening instrument for bipolar II disorder [104,105].

The PRISM and SCID-II interviews will be videotaped if the patients give their written consent. Ten randomly chosen diagnostic interviews will be rerated by an experienced clinician blind to the first interviewer's diagnosis. The information from PRISM, SCID-II, and IDS should make it possible to reclassify the symptoms later into the International Classification of Diseases and Related Health Problems (ICD-10) or newer versions of diagnostic manuals.

The PRISM interview includes present and lifetime diagnoses. The first occurrence and the lasting symptoms that qualify as SUD are registered. The duration of untreated SUD (DUSUD) can then be calculated.

The patients will be asked their reasons for starting to use these substances. Similar questions have been used in other studies. The general clinical impression of the patient and the diagnostic hypothesis will be described in text format, shortly after the diagnostic interviews. The patient will also be asked to sign a statement of agreement to be contacted for a follow-up study about 3-5 years after the first contact.

### Neurobiological assessment

To rule out undetected medical disorders that may be associated with psychiatric symptoms, blood samples will be analyzed for anemia, ongoing inflammation, blood, kidney, and liver diseases, thyroid and parathyroid diseases, diabetes, and vitamin B metabolic disorders. Carbohydrate-deficient transferrin together with liver enzymes will give further information about alcohol abuse or dependence disorders. The biological material will be included in the Bipolar Research and Innovation Network (BRAIN) biobank (see below) and will be analyzed for genetic molecular markers and cellular mechanisms underlying vulnerability to severe mental disorders.

### Estimation of sample size

The strength of this study lies not in the number of subjects but in the possibility of finding all subjects in a single catchment area who are consecutively admitted for treatment, and in the very thorough assessment of each subject. The estimation of sample size to ensure that statistically significant differences will be found between groups is problematic because earlier prevalence studies have varied to a large degree. The differences between the various groups of patients can only be estimated as qualified guesses. In this matter this study may be considered a pilot study for the creation of hypotheses for further studies.

### Statistical analysis

The Statistical Package for the Social Sciences version 16.0 will be used for all analyses. Statistical significance is defined at the 0.05 level with two-tailed tests of significance. The Chi squared test and Fisher's exact test will be used to investigate group differences in categorical data. Group differences in independent samples will be explored with Student's *t *test and analysis of variance (ANOVA) for normally distributed continuous variables and with the Mann-Whitney U test and Kruskal-Wallis test for variables with skewed distributions. The distributions of skewed variables will be presented as medians and interquartile ranges. Binary logistic regression analyses will be used to investigate the relationships between a dichotomous dependent variable and multiple dependent variables. Hierarchical multiple regression analyses will be used to investigate the relationships between one continuous dependent variable and multiple independent variables. Two-way ANOVA will be used to investigate possible interactions between variables.

## Discussion

### Methodological strengths

All Norwegian psychiatric and addiction services are public and available to everyone. Most patients with mental or addiction problems are referred to the psychiatric department of the local hospital for their catchment area. Patients referred to other hospitals or institutions will be identified through close contact with these hospitals, institutions, general practitioners (GPs), and social services. In this way, it will be possible to identify and include most of the patients from a single catchment area who meet the study criteria. By selecting a sample of first-time-admitted patients, we will avoid the overrepresentation of chronically ill patients. We will use robust and validated diagnostic and psychometric instruments. The main diagnostic interview will be undertaken to differentiate between substance-independent and substance-induced disorders. The interviewer will choose the time and place of the appointments, and if necessary will provide transport to ensure that the patients are able to complete the interviews. This will limit the number of dropouts. The Norwegian Data Inspectorate has permitted some basic (unidentifiable) data to be collected from those patients who refuse to participate in the study, to assess whether the sample is representative. The interviewer has taken part in PRISM training and interrater reliability training for the different assessment and rating scales. All the PRISM and SCID-II interviews will be performed by the same psychiatrist, so interrater reliability problems should be avoided. Some of the PRISM and SCID-II interviews will be videotaped (with the patients' written consent) and scored blindly by another qualified rater.

### Methodological limitations

Some of the patients will be users of psychoactive substances during the period of the interviews, so there will not be a four-week period of abstinence before the assessments. This may weaken the reliability of the information given by these patients. The study does not have a system for identifying all substance users in the catchment area. There will be some uncertainty about whether the treatment seekers are representative of the whole population. The sample size may be too small for the comparison of subgroups.

### Scientific implications

All patients with SUDs, admitted for the first time as inpatients or outpatients for psychiatric or addiction treatment, from a single catchment area during a specific time period, will be studied and diagnosed for substance use, and axis I and axis II comorbidity. As far as we know, this has not been done previously. At first-time admission, the first symptoms to occur and the disorder itself can be assessed more reliably than in later life, when this information must be reconstructed from memory. Therefore, this sample will make it possible to differentiate more accurately between independent and substance-induced disorders. Consequently, this study will better describe the prevalence of dual disorders than have most previous studies.

This study will be one of the first Norwegian studies to use the PRISM interview. A Norwegian version of this interview has recently been authorized. The interview is designed to diagnose comorbid substance-related and mental disorders. It is very important to acquire experience with the different recommended instruments within this field of clinical research.

### Clinical implications

For some decades now, attention has been directed to the complicated issue of diagnostic problems in patients with multiple disorders. It is extremely important to identify any independent psychiatric comorbidity in SUD patients and any comorbid SUDs in patients with mental disorders. Comorbidity seems to be the rule more often than the exception. In planning treatment, the following must be considered: the severity of the condition; whether the disorders are induced or independent; whether they should be treated separately, sequentially, or integrated; and where to find qualified treatment. An adequate diagnosis is necessary for this process. This study may show that the chosen assessment instruments are suitable. However, the interviews used in this study are time-consuming. It is probably not possible to perform this kind of diagnostic work in a time-efficient way.

Because the prevalence of these disorders varies widely between studies, it will be interesting to make a thorough diagnostic assessment of all first-time-admitted patients in a single catchment area, using a diagnostic interview which is proven to be reliable in dual disorder patients. More valid estimates of the prevalence of comorbidity in treatment seekers can then be presented. The catchment area based concept makes it possible to study a complete naturalistic sample, while most of the earlier studies have chosen convenience samples. This study of first time treatment seekers will avoid the problem of over representation of the most severely ill patients, and the retrospectively recalled symptoms will be less influenced by time lag and the effect of disease periods.

The duration of untreated SUD will be calculated. If the study shows that the duration of untreated SUD is long, e.g. several years, this will call for attention and better strategies for identifying SUD at an earlier stage. In many treatment settings for substance users, the skills in assessment and treatment of non substance mental disorders are limited. This is unproblematic if we find that most first time treatment seekers are mentally healthy except for their SUD, or if their mental disorders to a large extent are substance induced. If, however, this study reveals that most treatment seekers have comorbid disorders that demand specialized psychiatric treatment, today's treatment settings are insufficient. The division of patients with SUDs and psychiatric disorders into separate treatment clinics is based on tradition and not on professional consensus. This study may reveal new information that justifies either separate or combined services.

### Ethical considerations

This project is approved by the Regional Committee for Medical Research Ethics (registration number 6.2008.100), and by the Norwegian Data Inspectorate. The BRAIN study, including the biobank, has the necessary approvals. The project will be carried out according to the Declarations of Helsinki and Madrid.

None of the procedures used in this study presents any risk to the patients' health. All screening instruments and interviews are internationally acknowledged and validated. All of them have been used in previous studies in other projects worldwide.

Our common experience is that patients are not averse to being thoroughly examined and that they usually do not find the examinations too strenuous. All patients will be given oral and written information about the study before they give their written consent. If a patient refuses to take part in any of the assessments, this will be respected. If the patient refuses most of the interviews, it will be understood that he/she does not want to participate in the study. To test for bias, it will be necessary to record some basic, unidentifiable data about the patients who refuse to participate in the study; e.g., age, sex, and type of substances used.

It is possible that the biomedical or other examinations reveal information that makes the provision of adequate care necessary to avoid compromising the patient's health. With the patient's consent, such information will be passed on to the patient's therapist or GP. In some situations, the patients might not wish to inform their therapists or GPs. In such cases, the patients' wishes will be respected. If life-threatening depression, psychosis, or intoxication is identified, the patient will be referred for adequate treatment.

Patients between the ages of 16 and 18 years are considered able to give their full consent regarding their participation in a study of this kind. If the research fellow encounters problems that require treatment for which a young patient cannot give his/her consent, or the parents must be informed to exercise their parental responsibility, the patient's therapist or GP will be informed.

All patients of 18 years or older will be asked to agree to videotaped recordings of the interviews. Refusal will have no consequences for the patient. If the patient accepts, he/she will sign a separate statement of agreement. The purpose of the recording, the use of the videos, their safekeeping, and erasure will be described.

The patients will also be asked for their permission for the research fellow to contact them within 10 years to ask them to participate in a follow-up study. To take part in the follow-up study, they will sign a new written consent at the time of the follow-up. Refusal of this permission will have no consequences for the patient.

## List of abbreviations

ADHD: Attention deficit hyperactivity disorder; APA: American Psychiatric Association; AUDIT: Alcohol Use Disorder Identification Test; BRAIN: Bipolar Research and Innovation Network; DSM-IV: Diagnostic and Statistical Manual of Mental Disorders, Fourth Edition (APA); DUDIT: Drug Use Disorder Identification Test; DUSUD: Duration of Untreated Substance Use Disorder; GAF-F: Global Assessment of Functioning, Function Score; GAF-S: Global Assessment of Functioning, Symptom Score; GP: General practitioner; HCL-32: Angst Hypomania Check List, 32 Questions; IDS: Inventory of Depressive Symptoms; MADRS: Montgomery Asberg Depression Rating Scale; M.I.N.I.: the Mini International Neuropsychiatric Interview; NEQ: Stanley Foundation's Network Entry Questionnaire; NORMOOD: Norwegian Research Network on Mood Disorders; PRISM: Psychiatric Research Interview for Substance and Mental Disorders; SCID-II: Structured Clinical Interview for DSM-IV, axis II disorders; SCL-90R: Symptom Check List, 90 questions, Revised; SID: Substance-induced disorder; SUD: Substance use disorder (abuse or dependence of a psychoactive substance); TOP: Thematic Research Area Psychosis (Tematisk Område Psykose); YMRS: Young Mania Rating Scale

## Competing interests

The study is financed by governmental money and non commercial charity funds: Vestre Viken Hospital Trust, Psychiatric Department, Kongsberg; Innlandet Hospital Trust, Regional Center for Dual Diagnoses; University of Oslo, Institute of Clinical Medicine; Solveig and Johan P. Sommer's Foundation (non commercial charity research fund); Brukseier Jon Nielsen and Maja-Jonn Nielsen's Legacy (non commercial charity research fund). The activities of the NORMOOD research network are financed by Helse SørØst RHF (South-Eastern Norway Regional Health Authority).

AML declares that she has no competing interests. UFM has been given fees for lecturing by Astra Zeneca, Bristol-Myers Squibb, Glaxo Smith Kline, Lilly, Lundbeck, MSD (Organon), and Wyeth. His research group has been given an unrestricted research grant by Lundbeck. His spouse worked as a medical advisor for Pfizer Norway until 2010. SO has been given fees for lecturing by Bristol-Myers Squibb, as principal investigator for investigations sponsored by Astra Zeneca and Janssen Cilag, and as member of a Nordic expert group on antipsychotic drugs sponsored by Janssen Cilag. None of the pharmaceutical companies listed have any connection with or influence on the current project.

## Authors' contributions

UFM organized and secured the financial support for the study. All authors have contributed to the background, design, and drafting of the manuscript. All authors have read and approved the final manuscript.

## Pre-publication history

The pre-publication history for this paper can be accessed here:

http://www.biomedcentral.com/1471-244X/11/25/prepub
